# The Development of Artificial Intelligence in Hernia Surgery: A Scoping Review

**DOI:** 10.3389/fsurg.2022.908014

**Published:** 2022-05-26

**Authors:** Anas Taha, Bassey Enodien, Daniel M. Frey, Stephanie Taha-Mehlitz

**Affiliations:** ^1^ Department of Biomedical Engineering, Faculty of Medicine, University of Basel, Allschwil, Switzerland; ^2^ Department of Surgery, GZO- Hospital, Wetzikon, Switzerland; ^3^ Clarunis, Department of Visceral Surgery, University Center for Gastrointestinal and Liver Diseases, St. Clara Hospital and University Hospital, Basel, Switzerland

**Keywords:** scoping review, ventral hernia, artificial intelligence, inguinal hernia, incisional hernia

## Abstract

**Background:**

Artificial intelligence simulates human intelligence in machines that have undergone programming to make them think like human beings and imitate their activities. Artificial intelligence has dominated the medical sector to perform various patient diagnosis activities and improve communication between professionals and patients. The main goal of this study is to perform a scoping review to evaluate the development of artificial intelligence in all forms of hernia surgery except the diaphragm and upside-down hernia.

**Methods:**

The study used the Preferred Reporting Items for Systematic and Meta-analyses for Scoping Review (PRISMA-ScR) to guide the structuring of the manuscript and fulfill all the requirements of every subheading. The sources used to gather data are the PubMed, Cochrane, and EMBASE databases, IEEE and Google and Google Scholar search engines. AMSTAR tool is the most appropriate for assessing the methodological quality of the included studies.

**Results:**

The study exclusively included twenty articles, whereby seven focused on artificial intelligence in inguinal hernia surgery, six focused on abdominal hernia surgery, five on incisional hernia surgery, and two on AI in medical imaging and robotics in hernia surgery.

**Conclusion:**

The outcomes of this study reveal a significant literature gap on artificial intelligence in hernia surgery. The results also indicate that studies focus on inguinal hernia surgery more than any other types of hernia surgery since the articles addressing the topic are more. The study implies that more research is necessary for the field to develop and enjoy the benefits associated with AI. Thus, this situation will allow the integration of AI in activities like medical imaging and surgeon training.

## Introduction

### Rationale

Artificial intelligence simulates human intelligence in machines and prompts safe, accurate, and efficient solutions than traditional methods (1). Artificial intelligence applications include recommendation systems, search engines, autonomous vehicles, and speech recognition applications. According to Zhang and Lu, the coining of the term artificial intelligence occurred in 1956, and the concept has triggered multiple benefits for humanity by activating social development (2). The other advantages of artificial intelligence are that it guarantees enhanced efficiency by enabling the quicker performance of minimal and repetitive tasks. Unlike humans, machines cannot get tired of doing tasks that would be cumbersome to humans. Moreover, artificial intelligence ensures reduced human errors. Humans are susceptible to making errors compared to machines, especially when they are tired and vulnerable to committing mistakes. Consequently, the decisions derived via artificial intelligence are more factual than emotional, thus ensuring the derivation of quality and reasonable conclusions (3).

Artificial intelligence has dominated the medical sector to perform various activities like image processing and improved communication between professionals and patients through natural language processing (4). The other uses of artificial intelligence in the medical domain are end-to-end medicine innovation and development, transcription of medical documents like prescriptions, and treating patients from home. Briganti and Le Moine argued that despite the significant evolution of AI-powered technologies in the medical field, only specific settings benefit from applying the concept (5). They include epilepsy seizures, medical imaging for disease detection, and detecting atrial fibrillation (5). The main goal of this study is to evaluate the development of artificial intelligence in all forms of hernia surgery except the diaphragm and upside-down hernia. Hernia reflects an organ or flesh's bulging via an irregular opening, often self-diagnosable and treatable by healthcare professionals. The types of hernias this manuscript will address include the inguinal, abdominal, and incisional forms.

The researchers will incorporate a scoping review to gather the relevant information on the topic under study. This methodology aims to map the literature available on a particular topic and recognize any key concepts and gaps to inform practice and policymaking. The scoping review approach is suitable for answering the research question since integrating AI into hernia is still a new concept. Thus, the amount of literature available may be insufficient in giving conclusive evidence highlighting how the hernia domain has applied the notion. Through a scoping review, it is easier to gather an overview of the current knowledge in the field, highlight the gaps and guide future research on the subject (6). The approach's transparency and rigor also make it appropriate for this study.

### Objectives

The research question for this study is: what is the current state of AI integration in inguinal, abdominal, and incisional hernia surgeries?. The four research purposes delineated below will assist in inspecting the question further and replying to it. They include:

- To evaluate the current state of artificial intelligence in inguinal hernia surgery.
- To assess the current state and the anticipated future of artificial intelligence in abdominal hernia surgery.
- To examine artificial intelligence's current state and prospects in incisional hernia surgery.
- To offer recommendations for improving the development of artificial intelligence in hernia surgery.

## Methods

### Protocol and Registration

The protocol incorporated into this study was the Preferred Reporting Items for Systematic and Meta-analyses for Scoping Review (PRISMA-ScR). The methodology comprises twenty mandatory features and two optional elements that researchers engaging in a scoping review must adopt to ensure the quality of their results. This approach is suitable for this research. It will facilitate the evaluation of the study's advantages and shortcomings and permit the duplication of the review methods adopted by future researchers studying the topic. Also, PRISMA-ScR will guarantee the quality and transparency of the paper (7, 8). Sarkis-Onofre et al. argued that the technique allows authors to elaborate on the procedure undertaken in their study, the outcomes, and the plans (9). Thus, this protocol will enable the researcher to highlight their steps to derive their results.

### Eligibility Criteria

The eligibility criteria incorporated for this research depended on the relevancy of the articles. For instance, for inclusion, studies had to contain relevant information focusing on artificial intelligence in inguinal, abdominal, and incisional hernia surgeries. The researchers excluded any publications that concentrated on other hernia types. Also, the researcher ensured that the included articles comprised of original studies conducted on human participants to ensure the quality of the scoping review. Next, the researcher considered the publication dates of the relevant articles, whereby the authors included papers released between 2012 to 2022. The ten-year gap allowed the gathering of extensive information on the topic since the topic is still new and may not have sufficient supporting literature. Another factor considered by the researchers was language, where the authors included the articles in English and excluded any articles from foreign languages like French and German. This element ensured that the authors did not spend time translating the papers into other languages.

### Information Sources

Identifying the most appropriate documents for this study involved the researchers conducting a comprehensive literature search on various databases and search engines. The databases incorporated included PubMed, Cochrane, and EMBASE, whereas the search engines integrated were Google and Google Scholar. The authors explicitly looked for articles released from 2012 to 2022. The literature search took place for three days, from Mar 24, 2022, to Mar 27, 2022. A qualified librarian facilitated the process by detailing the search strategies to adopt during the literature search process. All the authors involved in the production of this manuscript collaborated to refine the adopted search strategies. A group discussion ensured that every researcher had an equal chance to air their views and concerns. Consequently, all two researchers scanned the references of relevant publications to acquire additional resources that qualified for inclusion in the study. The researchers also engaged in hand-searching of relevant journals to integrate into the study, which occurred in the Google and Google Scholar search engines. The integration of this approach allowed an extensive literature search, thereby ensuring the gathering of additional supporting information on the study topic.

### Search

The search strategy integrated into this research involved keying in the key terms in the relevant databases and search engines. On the PubMed, Cochrane, and EMBASE databases, the words used included “artificial intelligence,” “inguinal hernia surgery,” “abdominal hernia surgery,” and “incisional hernia surgery.” The filters incorporated on PubMed were “best match” and “2012 to 2022.” On Cochrane, the researchers used the date filter to ensure that the articles gathered were released from 2012 to 2022 and the trials filter to guarantee that the acquired publications were original studies. On EMBASE, we incorporated the “study type” and “publication year” filters to gather appropriate documents for inclusion in the study. On Google and Google Scholar search engines, the researchers keyed in phrases like “artificial intelligence in inguinal hernia surgery,” “artificial intelligence in abdominal hernia surgery,” and “artificial intelligence in incisional hernia surgery.” We did not use any filters on Google. However, on Google Scholar, the filters incorporated included “2012 to 2022,” “sort by relevance,” and “any type.” The oversight of the search process was the responsibility of the librarian who drafted the search strategy. The other authors peer-reviewed the method to guarantee validity and reliability by adopting the Peer Review of Electronic Search Strategies (PRESS) checklist. According to Bramer et al., the PRESS checklist guides researchers to check the search strategies integrated into a study (10). As a result, the approach allowed the authors to prove that the sources acquired reflected an accurate interpretation of the research question.

### Selection of Evidence Sources

Selecting evidence sources entailed contributing their efforts to the screening procedure. We screened all the acquired publications to ensure that they were full texts and contained the relevant information to qualify for inclusion. We also compared the abstracts and full texts to ensure that they were a match. We prevented instances where an included article lacked a complete text as it would be challenging to draw definitive conclusions. Before the screening, we first evaluated duplicate documents from all five avenues and eradicated them, reducing the workload during screening.

### Data Charting

We developed a data charting form and detailed all the factors to consider when acquiring information. The form highlighted elements like artificial intelligence and its use in inguinal, abdominal, and incisional hernia surgeries. We filled the data independently, after which they came together and discussed their findings. The updating of the form occurred iteratively. The authors resolved disagreements during this stage by re-evaluating the articles to achieve a non-biased consensus.

### Data Items

Data extraction relied on the various contextual factors of the articles. For instance, the papers addressed the medical domain and focused on developing artificial intelligence in inguinal, abdominal, and incisional hernia surgeries.

### Synthesis of Results

For this research, the authors grouped the publications based on their primary area of focus. The identified categories were inguinal hernia, abdominal hernia, and incisional hernia surgeries.

### Critical Appraisal of Results

The author incorporated the AMSTAR approach to examine the methodological quality adopted in the included studies, which enabled the solution of any disagreements between the two authors. The main advantage of the tool is that it ensures satisfactory reliability of various systematic reviews and randomized controlled treatment trials (11). The tool contains eleven items that researchers must assess to determine the quality of articles. This approach ensured that all the integrated studies fulfilled the requirements of the criterion.

## Results

### Selection of Evidence Sources

**Figure 1:**
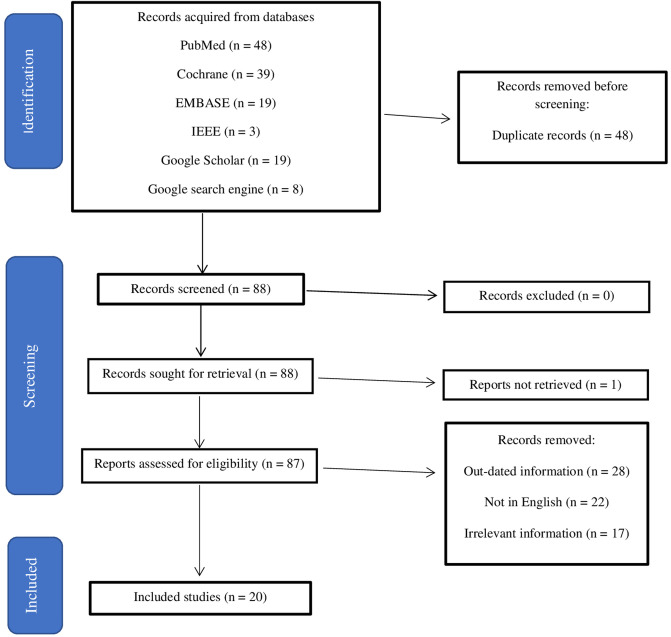
Inclusion and exclusion criteria for this study.

The search results on the PubMed, Cochrane, IEEE and EMBASE databases yielded 109 results, while Google Scholar and Google search engines produced twenty-seven results. The inclusion and exclusion criterion incorporated into the study is as shown in Figure 1.

### Study Characteristics

#### Results Synthesis

Out of the 20 included articles, seven (35%) focused on artificial intelligence in inguinal hernia surgery, six (30%) on artificial intelligence in abdominal hernia surgery, five (25%) on artificial intelligence in incisional hernia surgery, and two (10%) on AI in medical imaging and robotics in hernia surgery as displayed in Table 1. An overview of the main themes of the included research works gives Table 2.

**Table 1:** Main themes of included studies and characteristics of included papers.

Author	Years	Country	Primary Theme
AI in Inguinal Hernia Surgery			
Cui, Zhao, and Chen (12)	2021	China	CNN is effective in detecting vas deferens during inguinal hernia surgery
Gao, Zagadailov, and Merchant (13)	2021	USA	The Artificial Neural Network (ANN) is effective in detecting patient outcomes
O'Brien et al. (14)	2021	Multiple countries	The neural network model effectively predicts infection risks after inguinal hernia surgery.
Qin et al. (15)	2021	Multiple countries	Deep Neural Networks facilitate the hierarchical estimation of surgical states.
Ramshaw et al. (16)	2017	USA	Machine learning algorithms are vital in detecting wound infection during surgery.
Alonso-Silverio et al. (17)	2018	Mexico	A laparoscopic training system using artificial intelligence had the potential to increase trainee confidence.
Baloul et al. (18)	2020	USA	Machine Learning (ML) algorithms enhance the prediction of trainees’ training levels via video commentaries.
AI in Abdominal Hernia Surgery			
Muysoms et al. (19)	2012	Europe	AI application is evident since it facilitated the development of an online platform for registering and measuring ventral hernia surgery outcomes.
López-Cano et al. (20)	2021	Spain	AI application could enhance the quality of care given to abdominal hernia patients
Elhage et al. (21)	2021	China	Computed tomography images were more accurate than expert surgeons’ judgments in predicting surgical complexity.
Ramshaw (22)	2017	USA	AI could lead to sustainable healthcare
Friedrich et al. (23)	2019	Germany	NANEP model is effective in differentiating beginner and expert surgeons
Wang et al. (24)	2022	USA	An automated abdominal tissue classification algorithm attained extensive accuracy compared to previous algorithms.
AI in Incisional Hernia Surgery			
Kallinowski et al. (25)	2021	Multiple countries	Preoperative computed tomography was effective in predicting hernia recurrence.
Madani and Feldman (26)	2021	Multiple countries	AI, via ML, could assist in deriving intraoperative decisions.
Zipper et al. (27)	2020	Germany	AI is a practical approach for improving the technical abilities of surgeons.
Cole et al. (28)	2021	China	MI-CAIM approach showcased the potential for improving patient risk stratification
Licari et al. (29)	2019	Italy	The support vector model effectively predicted the risk factors triggering recurrences after incisional hernia surgery.
Robotics in Hernia Surgery			
Ozmen, Ozmen, and Koç (30)	2021	Switzerland	AI can enhance computer vision and prompt image-guided hernia surgeries
Donkor et al. (31)	2017	Europe	Robotics in hernia surgery provides opportunities for minimally-invasive surgeries while simultaneously reducing costs and length of hospital stay

**Table 2:** Data types, sizes, and evaluation metrics of included articles.

Author	Used Datatype	Dataset Size	Test Size	ML Type	Evaluation Metrics
Cui, Zhao, and Chen (12)	Videos from 35 inguinal surgery patients	2,600 images	1,200 images and 6 video clips	CNN	94.6% accuracy and 92.3% precision
Gao, Zagadailov, and Merchant (13)	Data from ACSNSQI Program	2,000 images	1,500 images	ANN	ANN and logistic regression evaluations were consistent
O'Brien et al. (14)	Veterans receiving hernia repair	96, 435 surgeries	40 patients	Neural Network Model	90% accuracy
Qin et al. (15)	RAS Dataset HERNIA -20	25,000 images	10 system events	HESS-DNN	80.4% accuracy
Ramshaw et al. (16)	CQI measurement after inguinal hernia repair	93 patients	93 patients	CQI model	48% of the patients showcased significant improvements
Alonso-Silverio et al. (17)	Two training sets	Four expert surgeons	Sixteen trainees	Laparoscopic Box Trainer system using AI and ANN	90.9% accuracy
Baloul et al. (18)	ML in the context of structured video commentary	13 short operative video clips	81 surgical residents	TensorFlow and Keras models	40% improvement
Muysoms et al. (19)	Online registry	Not applicable	Not applicable	3-dimensional numerical quality of life score	EuraHS website was effective
López-Cano et al. (20)	Explicit criteria for prioritizing waiting lists	92 patients	92 patients	AI explicit prioritization criteria	The criteria was accurate in separating waiting lists
Elhage et al. (21)	3 DLM models	369 patients	9,303 images	Computed tomography	81.3% accuracy compared to surgeon predictions
Ramshaw (22)	Assorted ML approaches	Not applicable	Not applicable-	Assorted AI models	The method demonstrated high accuracy
Friedrich et al. (23)	High-fidelity simulation approach	Internship students and experts	Internship students and experts	NANEP model	The model demonstrated significant accuracy levels
Wang et al. (24)	Fully automated abdominal tissue classifier algorithm	40 bovine and porcine biological sample	40 bovine and porcine biological sample	Multilayer Perception and CNN	91,14% accuracy and 91,06% precision
Kallinowski et al. (25)	Bench test	1 patient	1 patient	Computed tomography	The approach was accurate
Madani and Feldman (26)	Multiple machine learning approaches	Not applicable	Not applicable-	Multiple ML algorithms	Effectiveness of the approaches
Zipper et al. (27)	Two component silicones	6 beginners and 6 experts	6 beginners and six experts	High-fidelity model	Reliability of 81,1% to 97,4%
Cole et al. (28)	Clinical decision support tool	93,024 patients	93,024 patients	MI-CAIM	Model reduced operative time
Licari et al. (29)	Recurrence risk factors for incisional hernia surgery	154 patients	154 patients	Support vector model	86.2% Sensitivity and 86.6% accuracy
Ozmen, Ozmen, and Koç (30)	Various AI tools and Robots	Not applicable	Not applicable	Assorted AI models	Successful in hernia surgery
Donkor et al. (31)	Robotics in hernia surgery	Not applicable	Not applicable	Various robotic models	Achieved minimal invasion

## Discussion

### Summary of Evidence

#### Artificial Intelligence in Inguinal Hernia Surgery

Inguinal hernia surgery reflects the corrective procedure for correcting a bulge of tissues in the groin area. This type of surgery is one of the most prominent forms of repair performed globally. A study conducted by Cui, Zhao, and Chen aimed to test the effectiveness of the convolutional neural network (CNN) in detecting vas deferens (12). The study revealed that CNN recognized and labelled vas deferens images during laparoscopic inguinal hernia surgery (12). Another survey by Gao, Zagadailov, and Merchant showcased AI application in inguinal hernia surgery via the success of the artificial neural network (ANN) in predicting patient outcomes after undergoing a surgical procedure (13). More importantly, O'Brien et al. applied the Network Neural Model to predict long-term skin and soft tissue infection after hernia surgery (14). The researchers evaluated 96,435 surgeries, out of which 79.7% were inguinal. The results demonstrated the efficiency of the NNM in discriminating between those with and without infections by showing excellent calibration (14). From another perspective, Qin et al. praised the significance of deep neural networks in enhancing the hierarchical estimation of surgical states (15). Therefore, these arguments imply that the inguinal hernia surgery sector has taken advantage of artificial intelligence to strengthen processes and guarantee the delivery of quality care to patients by predicting and handling complications beforehand.

In another study, Ramshaw et al. evaluated the effectiveness of a computer-vision methodology borrowing from supervised learning and machine learning algorithms in detecting wounds after inguinal hernia surgery (16). The study outcomes indicated that the developed model showcased effectiveness in automatically detecting wound infections among patients undergoing surgery (16). From a different perspective, research conducted by Alonso-Silverio et al. revealed that AI applies to inguinal hernia surgery to develop a laparoscopic training system for offering online training to surgeons (17). The design incorporated python programming, ANN, and Raspberry Pi to conduct the training. The study results revealed that the system demonstrated the potential of enhancing surgeons' confidence and is relevant to applications with limited resources (17). In a similar viewpoint, Baloul et al. argued that AI, via a machine learning algorithm developed by the researchers, enhanced the prediction of the involved trainees’ post-graduate year levels (18). The algorithm effectively gauged the learners’ levels based on extensive assessments like video commentaries. Nonetheless, the authors posited that the approach required more studies and a more significant dataset to derive conclusive and replicable results (18). Therefore, this argument implies that more studies are essential to determine how inguinal hernia surgeons and trainers can apply artificial intelligence to their advantage.

#### Artificial Intelligence in Abdominal Hernia Surgery

Abdominal hernia surgery, also known as ventral hernia surgery, reflects the procedure of repairing a bulge of tissues through a weak point in the abdominal wall muscles. The integration of artificial intelligence in abdominal hernia surgery has been significant, but the concept is still in its infancy in the domain. A study by Muysoms et al. indicated that the integration of artificial intelligence dates back to as early as 2012, whereby the European Hernia Society board established an online platform for registering and measuring the outcomes for abdominal hernia surgeries (19). The development of the online platform ensured that surgeons could use it to guide their practices for dealing with ventral hernias (19). In another study, López-Cano et al. argued that the incorporation of artificial intelligence techniques could improve the delivery of patient care by combining and analyzing thousands of abdominal hernia cases and classifying them based on severity and prioritization (20). Artificial intelligence in abdominal hernia surgery is evident via the research conducted by Elhage et al., which aimed to apply three deep learning models to predict complexity and wound infections after an abdominal hernia procedure (21). The study outcomes demonstrated that the three image-based models, through computed tomography images, were effective in predicting surgical complexity and more accurate than expert surgeon judgment (21). These arguments imply that AI has the potential of enhancing multiple processes involved in abdominal hernia surgery.

More importantly, Ramshaw's study revealed that the integration of artificial intelligence in abdominal hernia surgery is minimal despite the various benefits it encompasses, like enhanced communication and the development of computer analytics for improving patient outcomes (22). The author also insisted that artificial intelligence can ultimately lead to sustainable healthcare, especially for abdominal hernia patients (22). In another study, Friedrich et al. tested the efficiency of the NANEP (Nabelhernien-Netzimplatation-Präperitonal), which in English is Umbilical hernia mesh implantation preperitoneal, in predicting the differences between beginner and expert surgeons (23). The study outcomes revealed that the approach was an inexpensive and straightforward simulation technique with high fidelity, which facilitates meeting the educational needs of surgeons. Furthermore, Wang et al. developed a fully automatic abdominal tissue classification algorithm using amalgam multilayer perceptron (MLP) and CNN classifier (24). This classifier achieved the highest accuracy compared to other previous algorithms with an accuracy of 91,14% and a precision of 91,06% (24). This argument implies that despite the low application of AI in abdominal hernia surgery, multiple opportunities are available, given that AI developers engage in prior comprehensive research.

#### Artificial Intelligence in Incisional Hernia Surgery

Incisional hernia surgery represents the procedure for rectifying protruding tissue at a recovering surgical scar site. The prevalence of incisional hernias has increased over the years, and its biggest trigger is the increment in obesity cases. Nevertheless, specialists have demonstrated efforts to introduce artificial intelligence into the sector to improve the quality of care and predict complications before they occur to prevent adverse consequences. For example, Kallinowski et al. tested the elasticity of tissues using preoperative computed tomography (25). The results indicated that the approach effectively predicted recurrent hernia since the clinical application of the system to ninety patients demonstrated success (25). In another study, Madani and Feldman claimed that machine learning algorithms in incisional hernia surgery allowed surgeons to make appropriate interoperation decisions that guarantee zero complications after hernia surgery (26). Moreover, Zipper et al. established a model for ensuring the proficiency-based training of surgeons, whereby the approach demonstrated high reliability and success in enhancing the technical capabilities of surgeons (27). The arguments presented above illustrate that integrating artificial intelligence in incisional hernia surgery will trigger positive outcomes and reduce instances of recurrences.

Additionally, Cole et al. argued that the Minimum Information about Clinical Artificial Intelligence Modelling (MI-CAIM) approach was an effective tool for examining patient risk factors and guiding patients on the approaches to undertake during incisional hernia surgery (28). More importantly, Licari et al. adopted a Support Vector Machine (SVM) to analyze the factors triggering incisional hernia recurrence among 154 patients who had undergone the procedure from 2007 to 2017 (29). The technique's performance was excellent since it demonstrated a sensitivity of 86.25% and an accuracy of 86.67% (29). Therefore, these statistics indicate that the technique was valid for grouping the identified thirty-four risk factors causing incisional hernia recurrence.

Further, the integration of artificial intelligence in hernia surgery has been through its abilities to facilitate medical imaging and robotics. For instance, a study by Ozmen, Ozmen, and Koç insisted that AI can enhance computer vision, whereby axial images occur via the incorporation of image acquisition and clarification applications that facilitate image-guided surgeries and computer-aided diagnosis (30). In a similar perspective, Donkor et al. argued that the adoption of robotics in hernia surgery provides opportunities for minimally-invasive surgeries while simultaneously reducing costs and length of hospital stay (31). Most importantly, it remains evident that the integration of artificial intelligence in medical imaging and robotics has increased over the years despite the challenges to its adoption. More studies in the field are necessary to ensure the development of AI systems that predict and help manage patient outcomes after undergoing hernia surgery.

### Limitations

The main limitation of this study is the lack of sufficient information on the topic under study. The literature search only led to the identification of twenty relevant documents to include in the study, implying that the field remains understudied despite its importance. Another limitation of this study is that it exclusively included articles published in English. This factor could have contributed to the low number of literatures found. There could have been some articles containing relevant information in other languages.

### Practical and Research Implications

The outcomes derived via this study are vital since they will inform hernia surgery by detailing the best machine learning algorithms for performing different processes like diagnosis and outcome predictions. The study implies that extensive research is necessary to educate hernia surgeons on the various ways they can apply and reap maximum benefits from artificial intelligence. Nonetheless, additional research in the sector is necessary.

## Conclusions

It remains evident that an extensive literature gap exists due to the minimal number of articles retrieved to include in the study. Also, the results indicate that previous studies have focused on inguinal hernia surgery compared to other hernia types. Future researchers studying the topic should focus on exploring the impacts and development of artificial intelligence in hernia surgery. Researchers should conduct original research by creating and testing AI algorithms to gather the multiple benefits they encompass. The results have indicated that AI application in inguinal, abdominal, and incisional hernia surgery is minimal due to the infancy of the concept in the field. Hence, future researchers should examine how AI can assist in medical imaging and the training of surgeons.
